# Higher anthocyanin intake is associated with a lower risk of non-alcoholic fatty liver disease in the United States adult population

**DOI:** 10.3389/fnut.2023.1265507

**Published:** 2023-11-06

**Authors:** Shuai Xiang, Yujing Li, Ying Li, Wenjun Pan, Xiaoqian Wang, Yun Lu, Shanglong Liu

**Affiliations:** ^1^Department of Gastrointestinal Surgery, Affiliated Hospital of Qingdao University, Qingdao, China; ^2^College of Basic Medical Sciences, China Medical University, Shenyang, China; ^3^Department of Pathology, The First Hospital of China Medical University, Shenyang, China; ^4^Department of Blood Transfusion, Affiliated Hospital of Qingdao University, Qingdao, China; ^5^Department of Nephrology, The Affiliated Hospital of Qingdao University, Qingdao, China

**Keywords:** flavonoid, anthocyanin, NAFLD, NHANES, liver fibrosis

## Abstract

**Background:**

Flavonoids are a class of plant chemicals known to have health-promoting properties, including six subclasses. Anthocyanin is one of the subclasses that have anti-inflammatory and antioxidant activities. However, the relationship between flavonoid subclass intake and the risk of non-alcoholic fatty liver disease (NAFLD) and liver fibrosis has not been verified in representative samples of the United States.

**Methods:**

This is a cross-sectional study based on the data from the National Health and Nutrition Examination Survey (NHANES) and the Food and Nutrient Database for Dietary Studies (FNDDS) in 2017–2018. The intake of flavonoid subclasses of the participants was obtained from two 24 h dietary recalls. The NAFLD and liver fibrosis were defined based on the international consensus criteria. The relationship between flavonoid subclass intake and NAFLD and liver fibrosis was evaluated using a multivariate logistic regression model corrected for multiple confounding factors. Subgroup analysis, trend tests, interaction tests and restricted cubic spline were carried out to further explore this relationship. In addition, we also explored the relationship between anthocyanin and liver serum biomarkers, dietary total energy intake and healthy eating index (HEI)-2015 scores.

**Results:**

A total of 2,288 participants were included in the analysis. The intake of anthocyanin was significantly negatively associated with the risk of NAFLD, but not other flavonoid subclasses. A higher anthocyanin intake was significantly associated with a lower risk of NAFLD (quartile 4, OR 0.470, 95% CI 0.275–0.803). The results of subgroup analysis showed that the protective effect of dietary anthocyanin intake on NAFLD was more pronounced in participants of non-Hispanic whites, with hypertension and without diabetes (*P* for interaction <0.05). Alanine aminotransferase (ALT), aspartate aminotransferase (AST), alkaline phosphatase (ALP), dietary total energy intake was significantly negatively correlated with dietary anthocyanin intake. We did not find any protective effect of flavonoid subclass intake on liver fibrosis.

**Conclusion:**

Anthocyanin, but not other flavonoid subclasses, can significantly reduce the risk of NAFLD. The protective effect was more pronounced in non-Hispanic whites, participants without diabetes and those with hypertension. Our study provides new evidence that anthocyanin intake has a reverse significant association with the risk for NAFLD.

## 1. Introduction

The prevalence of non-alcoholic fatty liver disease (NAFLD) has been rising steadily in recent years. In the United States, the number of NAFLD cases is expected to reach 100.9 million in 2030, increasing the burden on human health and society (1). NAFLD includes a number of liver diseases of varying severity and is defined as steatosis in more than 5% of liver cells in people who drink little or no alcohol (2). Metabolic disorder, oxidative stress, and local and systemic inflammation play important roles in the pathophysiology of NAFLD (3). Because the progression of NAFLD is continuous, liver cirrhosis and cancer can eventually occur. Although several drugs have shown curative effects in clinical trials, their long-term safety and effectiveness have not been verified due to the short clinical trial cycle (4).

Studies have shown that dietary components and micronutrients are closely related to NAFLD (5–7). Flavonoids are a class of natural compounds found in dietary components such as fruits, vegetables, red wine, and tea. Flavonoids contain a variety of subgroups including anthocyanin, flavan-3-ols, flavanones, flavones, flavonols, and isoflavones. Previous studies have found that flavonoids exert antioxidant activity and anti-inflammatory and anti-metabolic effects and have positive effects on lipid metabolism and insulin resistance. These pathways of action suggest that flavonoids are suitable for the treatment of NAFLD, and researchers have explored the potential of flavonoid use in preventing/treating NAFLD. Chang et al. showed that in the cell model of human hepatoma cell HepG2, mulberry anthocyanin extract (MAE) reduced blood lipids through phosphorylation of AMPK and inhibition of lipid biosynthesis, thus preventing NAFLD (8). Similarly, in HepG2 cells, Ou et al. found that mulberry extract could exert hypolipidemic effects by phosphorylation of AMPK and inhibition of lipid biosynthesis (9). In addition, in a NAFLD mouse model induced by a Western diet (WD), Nakano et al. found that providing bilberry anthocyanin in the diet could reduce the elevation of ALT and AST caused by the WD, and improve the intestinal microbiota dysbiosis, thus may be active in preventing NAFLD (10).

Previous studies on the health benefits of flavonoids have primarily been carried out in animal models (11–13), and few studies have been carried out on large representative samples. Mazidi et al. used the samples collected from the National Health and Nutrition Examination Survey (NHANES) from 2005 to 2010, and defined NAFLD patients as those with United States fatty liver index (USFLI) >30 (14). The results of multivariate logistic regression showed that flavonoid intake was significantly negatively associated with NAFLD risk. However, the authors did not explore which flavonoid subclass played a role in reducing NAFLD risk. Moreover, USFLI was used as the standard for NAFLD, which may lead to incorrect classification, as liver enzymes can be normal in some NAFLD cases (15). Compared with liver biopsy, ultrasound-based transient elastography of the liver is a non-invasive, objective measurement for liver fibrosis and liver steatosis, which has good specificity and sensitivity (16, 17).

Therefore, the purpose of this study was to explore the relationship between flavonoid subtypes and NAFLD and liver fibrosis using liver ultrasound-based transient elastography.

## 2. Methods

### 2.1. Study design and population

**Figure 1:**
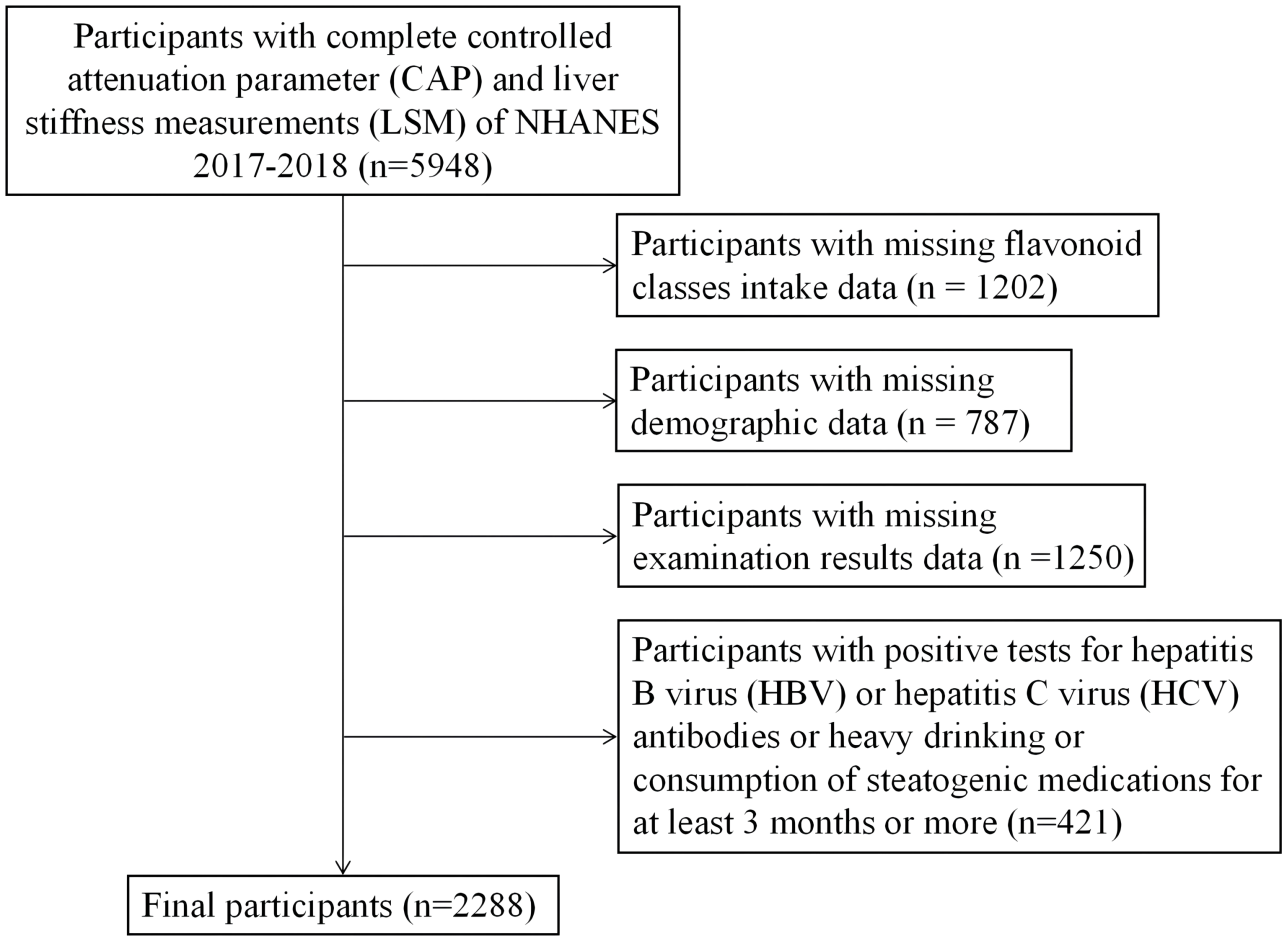
Research objectives selection process.

The NHANES is a cross-sectional population-based survey that adopted a stratified, multistage, and probability-cluster design to obtain a national representative sample of non-institutionalized civilians in the United States. Information such as demographics, socioeconomic status, diet, and health status was collected via questionnaire. Measures such as physical and laboratory examinations were obtained in the mobile examination unit (MEC). In this study, we extracted relevant NHANES data from 2017 to 2018. From these data, we identified 5,948 people with complete ultrasound-based liver transient elastography data, which were used to determine liver fibrosis and liver steatosis. Individuals with a positive hepatitis B surface antigen, positive hepatitis C antibody, heavy drinkers (more than 30 grams per day for men and more than 20 grams per day for women), taking amiodarone, corticosteroids, methotrexate, tamoxifen, sodium valproate, or other drugs known to be related to NAFLD for more than 6 months, and having missing data were excluded. Finally, 2,288 participants who met the requirements were included (Figure 1). The National Center for Health Statistics (NCHS) Research Ethics Review Board approved the NHANES research program, and informed consent was obtained from all participants. The NCHS Ethics Review Board (ERB) protocol number for NHANES 2017–2018 is Protocol #2018–01 (More information can be found in the website, https://www.cdc.gov/nchs/nhanes/irba98.htm).

### 2.2. Defining NAFLD and liver fibrosis

During the 2017–2018 survey cycle, liver stiffness measures (LSMs) were obtained by FibroScan^®^ using ultrasound and vibration controlled transient elastography (VCTE™). Ultrasound attenuation related to liver steatosis was measured at the same time, and the controlled attenuation parameter (CAP™) was recorded to evaluate liver steatosis (18). All participants aged 12 years and over were eligible. Participants were excluded if they (1) were unable to lie down on the exam table, (2) were pregnant (or unsure if pregnant) at the time of their exam, or a urine could not be obtained to test for pregnancy, (3) had an implanted electronic medical device, or (4) were wearing a bandage or had lesions on the right side of their abdomen by the ribs (where measurements would be taken). Based on the results of previous studies, we defined participants with a CAP ≥285 dB/m as NAFLD patients, and participants with an LSM ≥ 8.6 kPa as liver fibrosis patients (17). In 2016, a successful NCHS Ethics Review Board-approved pilot study of the liver ultrasound elastography examination demonstrated the following: (1) This non-invasive exam was safe and acceptable to over 400 NHANES participants who received it; (2) The exam performed well in the MEC; (3) Valid findings were obtained, namely the prevalence estimates of steatosis and fibrosis were similar for the two teams at two MEC locations and were similar to estimates in smaller communities published by others in the literature; (4) Valid findings were reported to approximately 90 percent of the participants who agreed to the examination.

### 2.3. Dietary intake

Detailed dietary intake information of NHANES participants was obtained through dietary recalls. The dietary intake data are used to estimate the types and amounts of foods and beverages (including all types of water) consumed during the 24 h period prior to the interview (midnight to midnight), and to estimate intakes of energy, nutrients, and other food components from those foods and beverages based on the USDA Food and Nutrient Database for Dietary Studies, 2.0 (FNDDS 2.0) (19). The first dietary recall was conducted in face-to-face interview, and the second was conducted by telephone interview 3–10 days later. The intakes of flavonoid classes, alcohol, total energy, protein, saturated fat, fiber, carbohydrates and polyunsaturated fat were calculated using the average of two 24 h dietary recalls.

### 2.4. Other covariates

Demographic variables included age, gender, race (divided into Non-Hispanic Black, Non-Hispanic White, Mexican American, and Other/Multi-Racial), education (including some college or above, high school graduate or GED, and less than high school graduate). Body Mass Index (BMI) was calculated as weight in kilograms divided by height in meters squared, and then rounded to one decimal place. Family income-to-poverty ratio (PIR) was calculated by dividing family (or individual) income by the poverty guidelines specific to the survey year. Smoking status was divided into former smokers (smoked more than 100 cigarettes in life and do not smoke at all now), never smokers (smoked less than 100 cigarettes in life), and current smokers (smoked more than 100 cigarettes in life and smoke regularly). Drinking status was divided into heavy drinkers (men drinking more than 30 g per day; women drinking more than 20 g per day), moderate drinkers (daily alcohol consumption for males between ≥20 g and < 30 g; female daily alcohol consumption between ≥10 g and < 20 g), mild drinkers (male daily alcohol consumption <20 g; women drinking less than 10 g a day), and never drinkers (0 g a day). Participants were diagnosed with diabetes if they met one of the following criteria: 1. doctor diagnosed diabetes; 2. glycohemoglobin HbA1c (%) > 6.5; 3. fasting glucose (mmol/l) ≥ 7.0; 4. random blood glucose (mmol/l) ≥ 11.1; 5. two-hour OGTT blood glucose (mmol/l) ≥ 11.1; 6. use of diabetes medications or insulin. Participants were considered to have hypertension if it was previously diagnosed by doctors, they were taking antihypertensive drugs, or had a systolic blood pressure ≥ 140 mmHg and/or diastolic blood pressure ≥ 90 mmHg. All blood pressure measurements (systolic and diastolic) were taken in the MEC and were taken in the right arm unless specific conditions prohibited the use of the right arm. After resting quietly in a seated position for 5 min, three consecutive blood pressure measurements (systolic and diastolic) were taken 60 s apart using a digital upper-arm electronic blood pressure measurement device, Omron HEM–907XL. The mean systolic or diastolic blood pressure was calculated as the average of three measurements. Physical activity (PA) information was self-reported by participants using the Global Physical Activity Questionnaire, which assesses the time spent doing different types of physical activity in a typical week, including vigorous and moderate intensity activities during work, transportation, and recreation. Then, we calculated the metabolic equivalent of task (MET) minutes using the conversion formula recommended by NHANES. Based on previous studies, we divided PA into <600, 600–7,999, ≥ 8,000 MET min/week (20). Using the latest version of healthy eating index (HEI) to evaluate diet quality, HEI-2015 scores ranged from 0 to 100, and the higher the score, the better the diet quality (21). HEI-2015 consists of 13 components, including total fruits, whole fruits, total vegetables, greens and beans, whole grains, dairy, total protein foods, seafood and plant proteins, fatty acids, refined grains, sodium, added sugars and saturated fats. Alanine aminotransferase (ALT), aspartate transferase (AST), alkaline phosphatase (ALP), high-sensitivity C-reactive protein (HS-CRP), high-density lipoprotein (HDL), fasting plasma glucose (FPG), triglyceride (TG), and total cholesterol (TC) levels were measured using a Roche Cobas 6,000 (c501 module) analyzer.

## 3. Statistical analysis

Classification variables are expressed as weighted percentages, and continuous variables are expressed as weighted averages (standard error). Differences in mean or percentage of baseline characteristics between NAFLD were tested by unadjusted binary logistic regression analysis. A weighted multivariate logistic regression analysis model was used to analyze the relationship between flavonoid subclass intake and NAFLD and liver fibrosis risk. Model 1 did not adjust for any covariates. Model 2 adjusted for age, sex, race, education level, PIR, BMI, smoking status, and alcohol use. Model 3 additionally adjusted for diabetes mellitus, hypertension, hyperlipidemia, HEI-2015 scores, total energy, dietary intakes of protein, saturated fat, fiber, carbohydrates, polyunsaturated fat and physical activity, based on Model 2. After adjusting for all covariates, weighted Restricted Cubic Spline (RCS) was used to fit the relationship between anthocyanin intake and NAFLD risk. To further explore the heterogeneity of the effect of anthocyanin on NAFLD risk, subgroup analysis was performed using age, race, BMI, sex, physical activity, DM, hypertension, hyperlipidemia, PIR, education level, smoking status, and alcohol use. The interaction between subgroups and anthocyanin intake was tested by incorporating the interaction item into the model using the likelihood ratio test. Moreover, a weighted multivariate linear regression analysis was used to explore the relationship between daily anthocyanidin intake and liver serum biomarkers, dietary total energy intake and HEI-2015 scores. All statistical analyses were performed in R (Version 4.2.2), and a bilateral *p* < 0.05 was considered statistically significant.

## 4. Results

### 4.1. Baseline characteristics

Of the 2,288 participants eventually included, 846 (37%) were diagnosed with NAFLD and 214 (9%) with liver fibrosis. NAFLD patients were older and more likely to be male and Mexican-American than non-NAFLD participants. Further, NAFLD patients were more likely to have a history of smoking, diabetes, hypertension, hyperlipidemia, less physical activity, and a high daily total energy intake, dietary protein intake and dietary carbohydrate intake. In addition, NAFLD patients also had increased BMI, ALT, AST, HS-CRP, FPG, TG, and TC and decreased HDL. Compared with the non-NAFLD population, NAFLD patients had a statistically lower intake of isoflavones and anthocyanin (Table 1).

**Table 1:** Characteristics of participants by the non-alcoholic fatty liver disease (NAFLD) status, weighted.

Variable	Total (*n*=2288)	Non-NAFLD (*n*=1442)	NAFLD (*n*=846)	*p*-value^a^
*Demographics*				
Age, mean (SE), y	46.75 (0.80)	44.48 (0.83)	50.89 (0.98)	**<0.0001**
Sex, *n* (%)				**0.03**
Female	1145 (50.04)	767 (52.84)	378 (45.21)	
Male	1143 (49.96)	675 (47.16)	468 (54.79)	
Race/ethnicity, *n* (%)				**<0.001**
Non-Hispanic Black	502 (21.94)	369 (11.74)	133 (7.04)	
Non-Hispanic White	841 (36.76)	510 (66.36)	331 (65.19)	
Mexican American	304 (13.29)	143 (5.87)	161 (12.17)	
Other/Multi-Racial	641 (28.02)	420 (16.03)	221 (15.60)	
Educational attainment, *n* (%)				**0.09**
Some college or above	1327 (58)	855 (64.76)	472 (58.78)	
High school graduate or GED	604 (26.4)	372 (26.54)	232 (32.32)	
Less than high school graduate	357 (15.6)	215 (8.69)	142 (8.91)	
BMI, kg/m^2^, *n* (%)				**<0.0001**
<25 kg/m^2^	613 (26.79)	567 (40.19)	46 (3.99)	
≥25 kg/m^2^	1675 (73.21)	875 (59.81)	800 (96.01)	
Smoking status, *n* (%)				**0.03**
Former	529 (23.12)	288 (21.25)	241 (29.86)	
Never	1413 (61.76)	927 (64.50)	486 (57.29)	
Current	346 (15.12)	227 (14.26)	119 (12.85)	
Alcohol use, *n* (%)				**0.24**
Mild	88 (3.85)	53 (4.25)	35 (4.66)	
Moderate	228 (9.97)	155 (13.74)	73 (9.76)	
Never	1972 (86.19)	1234 (82.00)	738 (85.58)	
Diabetes mellitus, *n* (%)				**<0.0001**
Yes	421 (18.4)	154 (6.31)	267 (26.72)	
No	1867 (81.6)	1288 (93.69)	579 (73.28)	
Hypertension, *n* (%)				**<0.0001**
Yes	938 (41)	464 (24.65)	474 (52.86)	
No	1350 (59)	978 (75.35)	372 (47.14)	
Hyperlipidemia, *n* (%)				**<0.0001**
Yes	1550 (67.74)	856 (59.05)	694 (81.90)	
No	738 (32.26)	586 (40.95)	152 (18.10)	
PIR, *n* (%)				**0.23**
<1.30	616 (26.92)	399 (18.59)	217 (17.28)	
1.30–3.49	932 (40.73)	562 (33.65)	370 (37.98)	
≥3.50	740 (32.34)	481 (47.76)	259 (44.74)	
Physical activity, MET min/week, *n* (%)				**<0.001**
<600	362 (15.82)	204 (10.20)	158 (19.41)	
600–7999	1392 (60.84)	892 (64.63)	500 (59.67)	
**≥**8000	534 (23.34)	346 (25.17)	188 (20.92)	
*Examination results, mean (SE)*				
ALT, U/L	22.44 (0.36)	19.51 (0.32)	27.78 (0.85)	**<0.0001**
AST, U/L	21.55 (0.24)	20.92 (0.32)	22.69 (0.40)	**0.01**
HS-CRP, mg/L	3.41 (0.20)	2.69 (0.21)	4.71 (0.26)	**<0.0001**
HDL, mg/dL	53.07 (0.45)	56.20 (0.48)	47.35 (0.46)	**<0.0001**
FPG, mg/dL	98.68 (0.80)	92.44 (0.39)	110.05 (1.85)	**<0.0001**
TG, mg/dL	138.62 (3.96)	113.45 (2.88)	184.54 (5.16)	**<0.0001**
TC, mg/dL	188.92 (1.86)	187.04 (1.76)	192.35 (2.73)	**0.04**
*Dietary measures, mean (SE)*				
Total energy, kcal/day	4119.81 (64.57)	4055.67 (82.82)	4236.82 (62.56)	**0.04**
Dietary protein, g/day	83.57 (1.73)	81.37 (2.14)	87.58 (1.61)	**0.004**
Dietary carbohydrate, g/day	250.13 (3.81)	244.51 (4.70)	260.39 (3.38)	**<0.001**
Dietary fiber, g/day	17.26 (0.49)	17.46 (0.68)	16.90 (0.52)	0.510
Dietary saturated fat, g/day	29.55 (0.46)	28.61 (0.68)	31.25 (0.67)	**0.02**
Dietary polyunsaturated fat, g/day	21.07 (0.51)	20.82 (0.64)	21.52 (0.57)	0.33
HEI-2015 scores	49.64 (0.78)	50.33 (0.97)	48.38 (1.03)	0.16
*Flavonoid classes intake, mg/day, mean (SE)*				
Total flavonoids	220.92 (12.52)	204.14 (13.71)	251.55 (27.89)	0.170
Isoflavones	2.60 (0.35)	3.12 (0.53)	1.65 (0.33)	**0.04**
Anthocyanidins	17.76 (1.99)	20.76 (2.48)	12.30 (1.48)	**<0.001**
Flavan_3_ols	171.11 (12.30)	151.31 (12.62)	207.25 (27.72)	0.1
Flavanones	10.10 (0.72)	10.22 (0.90)	9.88 (1.15)	0.82
Flavones	0.99 (0.06)	0.99 (0.07)	1.01 (0.08)	0.81
Flavonols	18.35 (0.49)	17.75 (0.71)	19.46 (1.04)	0.25
CAP, dB/m, mean (SE)	260.76 (2.35)	222.92 (1.27)	329.80 (1.77)	**<0.0001**
LSM, kPa, mean (SE)	5.85 (0.17)	4.87 (0.10)	7.65 (0.42)	**<0.0001**

SE, standard error; BMI, Body Mass Index; PIR, family income–to-poverty ratio; ALT, Alanine transaminase; AST, Aspartate transaminase; HS-CRP, hypersensitive C-reactive protein; HDL, high density lipoprotein; FPG, fasting plasma glucose; TG, triglyceride; TC, total cholesterol; HEI-2015 scores, healthy eating index-2015 scores; CAP, controlled attenuation parameter; LSM, liver stiffness measure.

a Unadjusted *p* values for comparison among the two groups. Bold values mean *p* < 0.05.

### 4.2. Participants with higher anthocyanin intake had a lower risk of NAFLD

We established a multivariate logistic regression model to explore the correlation between flavonoid subclasses and NAFLD and liver fibrosis. The results showed that five flavonoids (flavan-3-ols, flavanones, flavones, flavonols, and isoflavones) had no significant correlation with NAFLD risk (Supplementary Table S1). Anthocyanin showed a significant correlation after correcting for various covariates. None of the flavonoid subclasses were significantly associated with the risk of liver fibrosis. In Models 2 and 3, flavan_3_ol Q3 intake increased the risk of liver fibrosis compared with Q1 (Supplementary Table S2).

**Figure 2:**
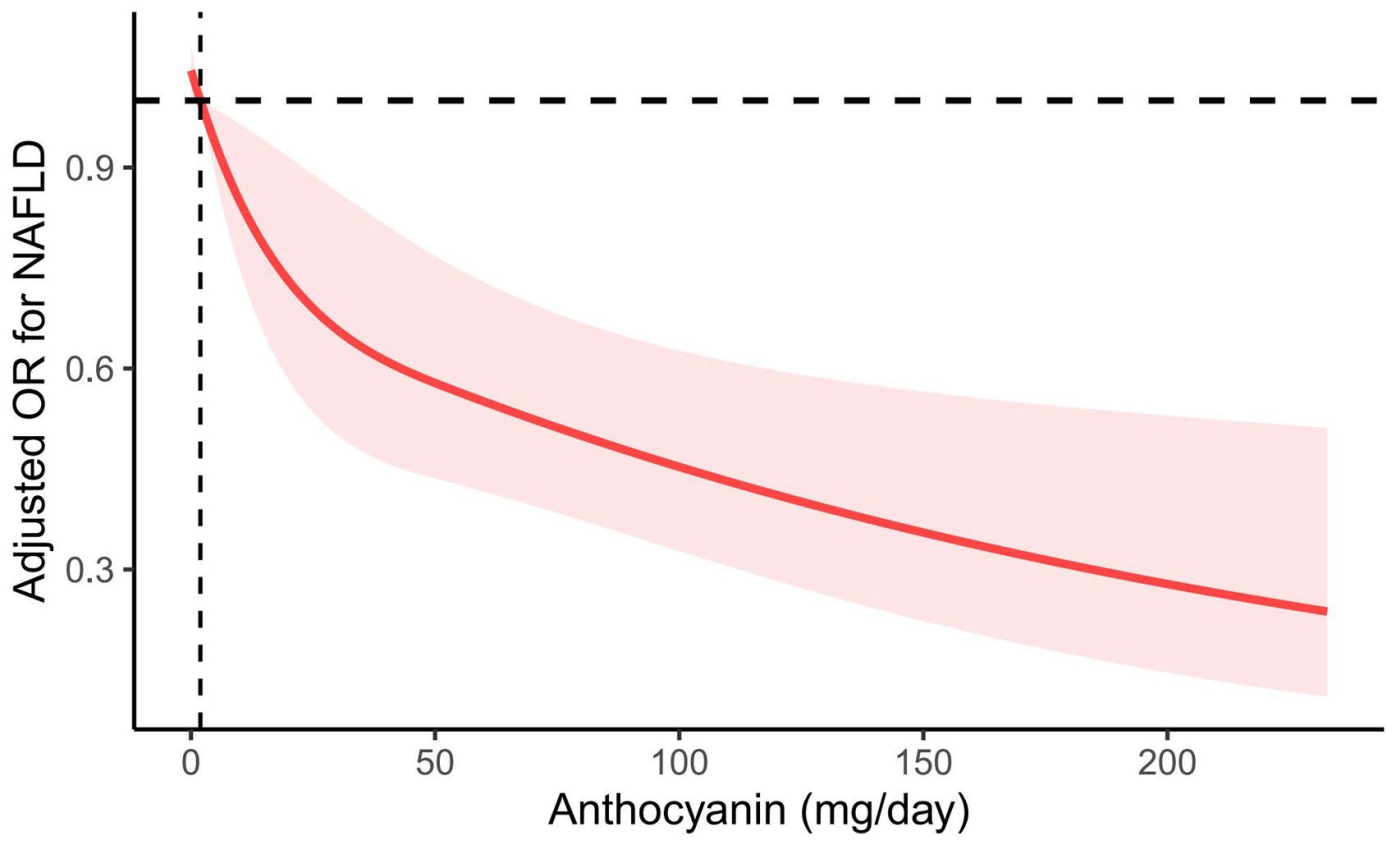
RCS plot between anthocyanin intake and the risk of NAFLD. Age, sex, race, education level, PIR, BMI, smoking status, alcohol use, diabetes mellitus, hypertension, hyperlipidemia, HEI-2015 scores, total energy, dietary intakes of protein, saturated fat, fiber, carbohydrates, polyunsaturated fat, and physical activity were adjusted. The solid line represents the estimated OR, and the red area represents the 95% CI.

However, anthocyanin intake was negatively correlated with NAFLD risk (Table 2). In Model 1 with no adjustment for variables, the highest level of anthocyanin intake (Q4) was negatively correlated with NAFLD (OR = 0.503, 95% CI, 0.359–0.705, *P* for trend <0.001). In the second model corrected for age, sex, race, education level, PIR, BMI, smoking status, and alcohol use, the highest level of anthocyanin intake (Q4) remained negatively correlated with NAFLD (OR = 0.463, 95% CI, 0.291–0.737, *P* for trend = 0.005). After further correction for diabetes mellitus, hypertension, hyperlipidemia, HEI-2015 scores, total energy, dietary intakes of protein, saturated fat, fiber, carbohydrates, polyunsaturated fat and physical activity, the highest level of anthocyanin intake (Q4) remained negatively correlated with NAFLD (OR = 0.470, 95% CI, 0.275–0.803, *P* for trend = 0.014). When RCS was used to fit the smooth curve, the results further revealed a negative correlation between anthocyanin intake and NAFLD risk (Figure 2).

**Table 2:** The univariate and multivariate logistic regression analysis results of association between anthocyanidins intake with the risk of non-alcoholic fatty liver disease (NAFLD) prevalence, weighted.

Anthocyanidins intake range (mg/day)	Model 1 [OR (95% CI)]	*p*-value	Model 2 [OR (95% CI)]	*p*-value	Model 3 [OR (95% CI)]	*p*-value
Quartile 1 [0, 0.06]	Referent		Referent		Referent	
Quartile 2 [0.06, 1.933]	0.834 (0.570, 1.221)	0.320	0.740 (0.480, 1.140)	0.158	0.691 (0.425, 1.123)	0.125
Quartile 3 [1.933, 13.561]	0.835 (0.596, 1.170)	0.267	0.753 (0.526, 1.077)	0.112	0.802 (0.544, 1.182)	0.244
Quartile 4 [13.561, 643.83]	0.503 (0.359, 0.705)	**<0.001**	0.463 (0.291, 0.737)	**0.003**	0.470 (0.275, 0.803)	**0.009**
*P* for trend	**<0.001**		**0.005**		**0.014**	
Continuously	0.994 (0.992, 0.997)	**<0.001**	0.993 (0.990, 0.997)	**<0.001**	0.993 (0.989, 0.996)	**<0.001**

OR, odds ratio; 95% CI, 95% confidence interval. Model 1: No covariates were adjusted. Model 2: Age, sex, race, education level, PIR, BMI, smoking status and alcohol use were adjusted. Model 3: Age, sex, race, education level, PIR, BMI, smoking status, alcohol use, diabetes mellitus, hypertension, hyperlipidemia, HEI-2015 scores, total energy, dietary intakes of protein, saturated fat, fiber, carbohydrates, polyunsaturated fat and physical activity were adjusted. Bold values mean *p* < 0.05.

We also performed a subgroup analysis based on age, sex, race, BMI, physical activity, DM, hypertension, hyperlipidemia, PIR, education level, smoking status, and alcohol use (Table 3). Results showed a stronger negative correlation between dietary anthocyanin intake and NAFLD risk in subgroup populations who were non-Hispanic whites, without diabetes mellitus and with hypertension (*P* for interaction<0.05).

**Table 3:** Subgroup analyses of the association between anthocyanidins intake and NAFLD diagnosed by vibration controlled transient elastography, weighted.

	Anthocyanidins intake (mg/day)					
	Quartile 1 [0, 0.06]	Quartile 2 [0.06, 1.933] OR (95% CI)	Quartile 3 [1.933, 13.561] OR (95% CI)	Quartile 4 [13.561, 643.83] OR (95% CI)	*P* for trend	*P* for interaction
**Age subgroup**						0.151
<70	Referent	0.694 (0.431, 1.118)	0.784 (0.515, 1.193)	0.528 (0.281, 0.991)	0.057	
>70	Referent	0.269 (0.061, 1.186)	0.482 (0.098, 2.382)	0.147 (0.030, 0.732)	0.072	
**Sex subgroup**						0.571
Female	Referent	1.043 (0.476, 2.286)	1.129 (0.482, 2.642)	0.779 (0.375, 1.617)	0.593	
Male	Referent	0.518 (0.301, 0.892)	0.602 (0.316, 1.147)	0.338 (0.169, 0.679)	**0.012**	
**Race subgroup**						**0.015**
Non-Hispanic Black	Referent	0.660 (0.287, 1.517)	1.868 (0.816, 4.275)	1.040 (0.494, 2.192)	0.267	
Mexican American	Referent	1.328 (0.454, 3.879)	1.203 (0.312, 4.642)	0.782 (0.326, 1.878)	0.367	
Non-Hispanic White	Referent	0.552 (0.277, 1.100)	0.740 (0.372, 1.470)	0.336 (0.152, 0.745)	**0.022**	
Other/Multi-Racial	Referent	1.252 (0.696, 2.253)	0.848 (0.368, 1.954)	0.881 (0.421, 1.846)	0.524	
**BMI subgroup**						0.330
<25 kg/m^2^	Referent	0.347 (0.052, 2.328)	0.618 (0.166, 2.301)	0.085 (0.019, 0.384)	**0.004**	
**≥25** kg/m^2^	Referent	0.753 (0.477, 1.191)	0.855 (0.581, 1.259)	0.540 (0.303, 0.962)	0.054	
**Physical activity subgroup**						0.305
<600	Referent	0.414 (0.135, 1.269)	0.811 (0.231, 2.845)	0.785 (0.238, 2.587)	0.908	
600–7999	Referent	0.784 (0.362, 1.695)	0.838 (0.488, 1.438)	0.400 (0.179, 0.898)	**0.024**	
**≥**8000	Referent	0.668 (0.301, 1.482)	0.683 (0.400, 1.165)	0.626 (0.252, 1.552)	0.289	
**Diabetes mellitus subgroup**						**0.016**
Yes	Referent	0.395 (0.152, 1.026)	1.061 (0.481, 2.340)	0.956 (0.382, 2.391)	0.917	
No	Referent	0.778 (0.470, 1.288)	0.767 (0.470, 1.253)	0.405 (0.209, 0.787)	**0.011**	
**Hypertension subgroup**						**0.036**
Yes	Referent	0.397 (0.238, 0.662)	0.726 (0.457, 1.151)	0.462 (0.279, 0.766)	**0.039**	
No	Referent	1.100 (0.519, 2.335)	0.953 (0.519, 1.751)	0.527 (0.214, 1.294)	0.131	
**Hyperlipidemia subgroup**						0.616
Yes	Referent	0.583 (0.324, 1.048)	0.722 (0.434, 1.202)	0.390 (0.212, 0.717)	**0.008**	
No	Referent	1.359 (0.691, 2.673)	1.257 (0.653, 2.420)	1.195 (0.560, 2.549)	0.683	
**PIR subgroup**						0.334
<1.3	Referent	0.880 (0.448, 1.726)	0.595 (0.341, 1.039)	0.628 (0.265, 1.487)	0.154	
1.3–3.49	Referent	0.926 (0.353, 2.426)	1.034 (0.513, 2.087)	0.385 (0.240, 0.617)	**0.002**	
**≥**3.49	Referent	0.381 (0.165, 0.881)	0.687 (0.314, 1.502)	0.440 (0.148, 1.315)	0.279	
**Education level subgroup**						0.441
Less than high school graduate	Referent	0.674 (0.285, 1.590)	0.994 (0.495, 1.998)	0.419 (0.126, 1.386)	0.323	
Some college or above	Referent	0.846 (0.460, 1.556)	0.742 (0.414, 1.329)	0.562 (0.286, 1.106)	0.063	
High school graduate or GED	Referent	0.365 (0.134, 0.997)	0.768 (0.313, 1.888)	0.371 (0.112, 1.229)	0.203	
**Smoking status subgroup**						0.445
Former	Referent	0.427 (0.172, 1.062)	0.886 (0.435, 1.805)	0.308 (0.105, 0.904)	0.131	
Never	Referent	0.730 (0.385, 1.386)	0.822 (0.386, 1.752)	0.527 (0.304, 0.912)	0.053	
Current	Referent	1.437 (0.618, 3.342)	0.372 (0.118, 1.177)	0.427 (0.120, 1.510)	0.075	
**Alcohol use subgroup**						0.407
Mild	Referent	0.610 (0.355, 1.048)	0.783 (0.466, 1.315)	0.492 (0.264, 0.915)	0.062	
Moderate	Referent	1.370 (0.188, 9.957)	1.873 (0.186, 18.862)	0.911 (0.183, 4.543)	0.926	
Never	Referent	0.550 (0.311, 0.970)	0.744 (0.451, 1.226)	0.513 (0.293, 0.896)	0.349	

Age, sex, race, education level, PIR, BMI, smoking status, alcohol use, diabetes mellitus, hypertension, hyperlipidemia, HEI-2015 scores, total energy, dietary intakes of protein, saturated fat, fiber, carbohydrates, polyunsaturated fat and physical activity were adjusted. Bold values mean *p* < 0.05.

### 4.3. Relationship between liver serum biomarkers, dietary total energy intake, HEI-2015 scores and dietary anthocyanin intake

We performed multivariate linear regression analysis of daily anthocyanin intake and liver serum biomarkers, dietary total energy intake and HEI-2015 scores (Table 4). The results showed that participants with a higher anthocyanin intake had lower plasma concentrations of ALT, AST, ALP, and dietary total energy intake.

**Table 4:** Multivariate linear regression analysis of daily anthocyanidins intake and liver serum biomarkers, dietary total energy intake and HEI-2015 scores.

	Anthocyanidins intake (mg/day)	
	β (95% CI)	*p*-value
Liver serum biomarkers		
ALT, U/L	−0.019 (−0.034, −0.005)	**0.012**
AST, U/L	−0.010 (−0.018, −0.002)	**0.019**
ALP, U/L	−0.030 (−0.057, −0.003)	**0.033**
HS-CRP, mg/L	−0.003 (−0.007, 0.001)	0.105
HDL, mg/dL	0.007 (−0.011, 0.024)	0.428
FPG, mg/dL	−0.006 (−0.031, 0.019)	0.593
TG, mg/dL	−0.016 (−0.147, 0.115)	0.802
TC, mg/dL	0.006 (−0.039, 0.051)	0.781
Total energy, kcal/day	−0.205 (−0.294, −0.116)	**<0.001**
HEI-2015 scores	0.023 (−0.009, 0.054)	0.143

Age, sex, race, education level, PIR, BMI, smoking status, alcohol use, diabetes mellitus, hypertension, hyperlipidemia, HEI-2015 scores, total energy, dietary intakes of protein, saturated fat, fiber, carbohydrates, polyunsaturated fat and physical activity were adjusted. Bold values mean *p* < 0.05.

## 5. Discussion

In our study, the weighted prevalence of NAFLD was 37%. The increasing incidence of NAFLD has had a negative effect on public health and has increased social burden. However, due to its complex pathogenesis, there are currently no drugs approved for the treatment of NAFLD and/or the related cirrhosis. Dietary intervention, physical activity, and exercise are considered the cornerstones of NAFLD/NASH treatment. Therefore, increasing attention has been paid to the primary prevention of NAFLD through improvements in diet and living habits (22). Our study found that higher levels of anthocyanin intake were significantly associated with lower NAFLD risk, while other flavonoid subtypes were not. In addition, no significant correlation was observed between all flavonoid subtypes and the risk of liver fibrosis. The protective effect of higher levels of anthocyanin was more significant in patients who were non-Hispanic whites, without diabetes mellitus and with hypertension. We also found that ALT, AST, and total energy intake decreased significantly in individuals with a high anthocyanin intake.

The classic “double blow theory” has been used to explain the pathogenesis of NAFLD (23). The first blow is steatosis caused by insulin resistance, which leads to the accumulation of fatty acids in the liver. Subsequently, liver lipid metabolism disorder leads to the activation of oxidative stress, the release of pro-inflammatory factors, and finally liver injury and fibrosis (24). Anthocyanin belongs to a subset of polyphenols called flavonoids. They are soluble in water and widely exist in fruits, grains, and vegetables, making them appear red-orange to blue-purple (25). *In vivo* and *in vitro* studies have found that anthocyanin can reduce the risk of NAFLD through various mechanisms. Kim et al. found that anthocyanin extracted from black chokeberry enhanced the expression of LDL-R mRNA and protein in Caco-2 cells and stimulated cholesterol transport (26). Chu et al. found that cherry anthocyanin prevented oxidative stress induced by oleic acid (OA-) and decreased the accumulation of lipid droplets by activating autophagy in a NAFLD cell model (27). Fan et al. found that polymeric anthocyanin extracted from grape skins inhibited oxidative stress by increasing antioxidant levels and increased β-oxidation to inhibit mitochondrial dysfunction on a NAFLD model in mice (28). A recent case–control and intervention study showed that after 12 weeks of anthocyanin capsules (320 mg per day) or placebo, the mRNA expression of NLRP3 inflammasome components in peripheral blood mononuclear cells (PBMCs) and the plasma levels of IL-1β and IL-18 were significantly reduced in NAFLD patients in the intervention group compared with the control group (29).

However, focused studies on the role of anthocyanin in the human body remain limited. This cross-sectional study found that dietary anthocyanin intake was significantly negatively correlated with ALT, AST, ALP and total energy intake using linear regression models adjusted for multiple confounders. Elevated levels of ALT, AST, and ALP are the most common abnormal findings in liver function tests, indicating increased permeability and damage of hepatocytes, and are usually considered as markers of NAFLD (30, 31). This is similar to the results of some previous studies. Mazidi et al. found that the higher the flavonoid intake, the more favorable the liver marker characteristics (manifested as significant reductions in AST and ALT) (14). A 12 weeks randomized controlled trial by Zhang et al. showed that ALT, cytokeratin-18 (M30 levels), and myeloperoxidase levels in participants taking purified anthocyanin (320 mg/d) decreased significantly compared with the placebo group (32). Moreover, higher total energy intake, along with excessive intake of ultra-processed foods and saturated fats, has long been considered as the main driving factor for NAFLD development (33). This study showed that participants with higher dietary anthocyanin intake had lower total energy intake, which may also be a reason for the reduced risk of NAFLD.

In the subgroup analysis, we found that some groups were more likely to benefit from anthocyanin intake. The United States is a multi-ethnic country, and our research found that anthocyanin only play a significant role in non-Hispanic whites. This may be due to differences in lifestyle, eating habits, and genetic background among races (34). It is well known that diabetes mellitus is independent risk factor for the occurrence and development of NAFLD (35). Patients with diabetes have higher levels of metabolic dysfunction and oxidative stress, which lead to more severe hepatic steatosis, inflammation and fibrosis, and impair their response to anthocyanin (36). Patients with diabetes may require higher doses or longer duration of anthocyanin treatment to achieve effective results. Moreover, anthocyanins have more pronounced effects in NAFLD patients with hypertension. Some preclinical studies have shown that anthocyanin have potential antihypertensive activity, which may be mediated by modulating endothelial nitric oxide synthase (eNOS) expression, antioxidant activity, inhibiting angiotensin-converting enzyme (ACE) activity and other pathways in endothelial cells (37). Anthocyanin may reduce liver damage by improving vascular tone and hemodynamics, lowering blood pressure and portal pressure, and may have special benefits for NAFLD patients with hypertension. These conclusions need more clinical trials to verify and support.

The results showed that except for anthocyanin, the other five flavonoid subclasses had no significant correlation with NAFLD risk. Yang et al.’s meta-analysis results showed that other polyphenols also had beneficial effects on NAFLD, including curcumin, catechin, and silymarin (38). However, due to only one randomized clinical trial (RCT) study on anthocyanin, the efficacy of anthocyanin could not be assessed. Therefore, this cross-sectional study partially filled this knowledge gap. In addition, all six flavonoids were not related to the risk of liver fibrosis. Since liver fibrosis is a manifestation of NAFLD progression, flavonoids may not be able to significantly improve the condition of patients at this stage.

Using representative, national sample data and after correcting for confounding factors, we explored the relationship between anthocyanin intake and NAFLD risk. We also conducted subgroup analyses, trend tests, and interaction tests to analyze the effects of anthocyanin in different individual groups. However, this study also has some limitations. First, the diet and physical activity data were reported by participants independently, which may have resulted in measurement errors. For this reason, we used the average of two 24 h dietary recalls to estimate flavonoid intake and minimize deviations. Second, although we corrected for many confounding factors, unidentified confounding factors may remain that could affect our conclusions. Third, more *in vivo* studies are needed to explore the specific mechanisms of anthocyanin in reducing the risk of NAFLD. Finally, because this was a cross-sectional study, we could identify a significant negative correlation between anthocyanin intake and NAFLD risk but could not determine a causal relationship.

## 6. Conclusion

Overall, this study provides new evidence that anthocyanin may significantly reduce the risk of NAFLD in the United States. The negative correlation was more significant among participants belonging to the following categories: non-Hispanic whites, without diabetes and with hypertension. In the future, a more elaborate experimental design is needed to clarify the minimum intake required to achieve health benefits.

## Data availability statement

The original contributions presented in the study are included in the article/Supplementary material, further inquiries can be directed to the corresponding authors.

## Ethics statement

The studies involving humans were approved by the NCHS Research Ethics Review Board approved the NHANES research program, and informed consent was obtained from all participants. The studies were conducted in accordance with the local legislation and institutional requirements. The participants provided their written informed consent to participate in this study.

## Author contributions

SX: Conceptualization, Data curation, Formal analysis, Methodology, Project administration, Writing – original draft. YujL: Data curation, Methodology, Writing – original draft. YiL: Conceptualization, Data curation, Methodology, Writing – review & editing. WP: Data curation, Formal analysis, Writing – original draft. XW: Data curation, Methodology, Writing – review & editing. YunL: Data curation, Methodology, Software, Supervision, Writing – review & editing. SL: Data curation, Methodology, Supervision, Writing – original draft, Writing – review & editing.
