# Supplementary material for: Higher anthocyanin intake is associated with a lower risk of non-alcoholic fatty liver disease in the United States adult population
Source: Front Nutr. 2023 Nov 6;10:1265507. doi: 10.3389/fnut.2023.1265507 (PMC10657849; doi:10.3389/fnut.2023.1265507)
Supplement: Supplementary file 1 [file Table_1.docx]

**TableS1 The univariate and multivariate logistic regression analysis results of association between Flavonoid classes intake in addition to anthocyanidins with the risk of non-alcoholic fatty liver disease (NAFLD) prevalence, weighted.**

| **Variables** | **Quartile 1** | **Quartile 2** | **Quartile 3** | **Quartile 4** | **P-trend** |
| --- | --- | --- | --- | --- | --- |
| **Isoflavones** |  |  |  |  |  |
| **Range (mg/day)** | [0, 0] | (0, 0.015] | (0.015, 0.15] | (0.15, 390.6] |  |
| **Model 1 [OR (95% CI) (P-value)]** | Referent | 1.099 (0.834, 1.450) (0.470) | 0.957 (0.620, 1.477) (0.829) | 0.719 (0.451, 1.146) (0.149) | 0.124 |
| **Model 2 [OR (95% CI) (P-value)]** | Referent | 1.286 (0.883, 1.875) (0.175) | 1.014 (0.632, 1.627) (0.951) | 0.907 (0.534, 1.538) (0.698) | 0.610 |
| **Model 3 [OR (95% CI) (P-value)]** | Referent | 1.254 (0.867, 1.813) (0.211) | 1.109 (0.674, 1.824) (0.664) | 0.901 (0.559, 1.451) (0.647) | 0.656 |
| **Flavan_3_ols** |  |  |  |  |  |
| **Range (mg/day)** | [0, 4.704] | (4.704, 16.94] | (16.94, 147.24] | (147.24, 4274.04] |  |
| **Model 1 [OR (95% CI) (P-value)]** | Referent | 1.318 (0.889,1.954) (0.152) | 1.291 (0.869, 1.919) (0.185) | 1.034 (0.723, 1.478) (0.844) | 0.931 |
| **Model 2 [OR (95% CI) (P-value)]** | Referent | 1.408 (0.886, 2.238) (0.136) | 1.549 (0.927, 2.589) (0.089) | 1.124 (0.710, 1.779) (0.597) | 0.578 |
| **Model 3 [OR (95% CI) (P-value)]** | Referent | 1.503 (0.931, 2.426) (0.090) | 1.663 (0.933, 2.965) (0.080) | 1.181 (0.692, 2.019) (0.517) | 0.599 |
| **Flavanones** |  |  |  |  |  |
| **Range (mg/day)** | [0, 0.01] | (0.01, 0.28] | (0.28, 12.672] | (12.672, 393.35] |  |
| **Model 1 [OR (95% CI) (P-value)]** | Referent | 1.102 (0.718, 1.692) (0.629) | 0.901 (0.649, 1.251) (0.504) | 1.097 (0.715, 1.683) (0.647) | 0.952 |
| **Model 2 [OR (95% CI) (P-value)]** | Referent | 1.103 (0.700, 1.738) (0.653) | 0.910 (0.578, 1.434) (0.666) | 1.045 (0.650, 1.679) (0.847) | 0.910 |
| **Model 3 [OR (95% CI) (P-value)]** | Referent | 1.210 (0.759, 1.928) (0.397) | 0.965 (0.613, 1.519) (0.868) | 1.127 (0.673, 1.887) (0.629) | 0.885 |
| **Flavones** |  |  |  |  |  |
| **Range (mg/day)** | [0, 0.155] | (0.155, 0.505] | (0.505, 1.265] | (1.265, 72.825] |  |
| **Model 1 [OR (95% CI) (P-value)]** | Referent | 0.946 (0.682, 1.311) (0.716) | 0.818 (0.514, 1.300) (0.363) | 0.957 (0.614, 1.492) (0.834) | 0.701 |
| **Model 2 [OR (95% CI) (P-value)]** | Referent | 0.859 (0.590, 1.252) (0.403) | 0.763 (0.424, 1.374) (0.343) | 0.994 (0.578, 1.710) (0.982) | 0.896 |
| **Model 3 [OR (95% CI) (P-value)]** | Referent | 0.941 (0.631, 1.403) (0.750) | 0.853 (0.477, 1.523) (0.567) | 1.026 (0.616, 1.710) (0.915) | 0.961 |
| **Flavonols** |  |  |  |  |  |
| **Range (mg/day)** | [0, 6.819] | (6.819, 12.535] | (12.535, 22.669] | (22.669, 207.145] |  |
| **Model 1 [OR (95% CI) (P-value)]** | Referent | 1.127 (0.820, 1.548) (0.428) | 0.914 (0.667, 1.251) (0.543) | 0.963 (0.611, 1.517) (0.859) | 0.610 |
| **Model 2 [OR (95% CI) (P-value)]** | Referent | 1.018 (0.676, 1.532) (0.928) | 0.836 (0.568, 1.232) (0.341) | 0.920 (0.568, 1.489) (0.717) | 0.527 |
| **Model 3 [OR (95% CI) (P-value)]** | Referent | 0.980 (0.598, 1.606) (0.931) | 0.813 (0.524, 1.263) (0.333) | 0.877 (0.491, 1.566) (0.637) | 0.483 |

**Notes: OR, odds ratio; 95% CI, 95% confidence interval. Model 1: No covariates were adjusted. Model 2: Age, sex, race, education level, PIR, BMI, smoking status, alcohol use were adjusted. Model 3: Age, sex, race, education level, PIR, BMI, smoking status, alcohol use, diabetes mellitus, hypertension, hyperlipidemia, HEI-2015 scores, total energy, dietary intakes of protein, saturated fat, fiber, carbohydrates, polyunsaturated fat and physical activity were adjusted.**
