# Supplementary material for: Higher anthocyanin intake is associated with a lower risk of non-alcoholic fatty liver disease in the United States adult population
Source: Front Nutr. 2023 Nov 6;10:1265507. doi: 10.3389/fnut.2023.1265507 (PMC10657849; doi:10.3389/fnut.2023.1265507)
Supplement: Supplementary file 2 [file Table_2.docx]

**TableS2 The univariate and multivariate logistic regression analysis results of association between Flavonoid classes intake with the risk of clinically significant fibrosis (CSF) prevalence, weighted.**

| **Variables** | **Quartile 1** | **Quartile 2** | **Quartile 3** | **Quartile 4** | **P-trend** |
| --- | --- | --- | --- | --- | --- |
| **Anthocyanins** |  |  |  |  |  |
| **Range (mg/day)** | [0, 0.06] | (0.06, 1.933] | (1.933, 13.561] | (13.561, 643.83] |  |
| **Model 1 [OR (95% CI) (P-value)]** | Referent | 0.967 (0.546, 1.711) (0.900) | 0.937 (0.487, 1.802) (0.832) | 0.702 (0.360, 1.371) (0.272) | 0.253 |
| **Model 2 [OR (95% CI) (P-value)]** | Referent | 0.971 (0.525, 1.795) (0.920) | 0.936 (0.481, 1.821) (0.835) | 0.796 (0.373, 1.699) (0.531) | 0.515 |
| **Model 3 [OR (95% CI) (P-value)]** | Referent | 0.910 (0.477, 1.735) (0.759) | 0.987 (0.495, 1.967) (0.968) | 0.719 (0.393, 1.315) (0.262) | 0.283 |
| **Isoflavones** |  |  |  |  |  |
| **Range (mg/day)** | [0, 0] | (0, 0.015] | (0.015, 0.15] | (0.15, 390.6] |  |
| **Model 1 [OR (95% CI) (P-value)]** | Referent | 0.939 (0.468, 1.885) (0.848) | 0.607 (0.283, 1.301) (0.179) | 0.612 (0.304, 1.231) (0.152) | 0.115 |
| **Model 2 [OR (95% CI) (P-value)]** | Referent | 1.075 (0.573, 2.019) (0.809) | 0.661 (0.328, 1.334) (0.228) | 0.770 (0.424, 1.397) (0.364) | 0.229 |
| **Model 3 [OR (95% CI) (P-value)]** | Referent | 0.946 (0.430, 2.079) (0.882) | 0.538 (0.255, 1.135) (0.097) | 0.720 (0.377, 1.376) (0.297) | 0.154 |
| **Flavan_3_ols** |  |  |  |  |  |
| **Range (mg/day)** | [0, 4.704] | (4.704, 16.94] | (16.94, 147.24] | (147.24, 4274.04] |  |
| **Model 1 [OR (95% CI) (P-value)]** | Referent | 0.934 (0.593, 1.474) (0.751) | 1.402 (0.919, 2.139) (0.107) | 0.990 (0.528, 1.856) (0.973) | 0.704 |
| **Model 2 [OR (95% CI) (P-value)]** | Referent | 0.965 (0.592, 1.573) (0.877) | 1.669 (1.101, 2.531) **(0.019)** | 1.155 (0.629, 2.120) (0.621) | 0.306 |
| **Model 3 [OR (95% CI) (P-value)]** | Referent | 0.975 (0.642, 1.481) (0.900) | 1.725 (1.226, 2.427) **(0.004)** | 1.153 (0.697, 1.908) (0.557) | 0.253 |
| **Flavanones** |  |  |  |  |  |
| **Range (mg/day)** | [0, 0.01] | (0.01, 0.28] | (0.28, 12.672] | (12.672, 393.35] |  |
| **Model 1 [OR (95% CI) (P-value)]** | Referent | 1.308 (0.597, 2.864) (0.470) | 1.021 (0.536, 1.946) (0.945) | 1.338 (0.609, 2.940) (0.435) | 0.540 |
| **Model 2 [OR (95% CI) (P-value)]** | Referent | 1.409 (0.660, 3.006) (0.351) | 1.186 (0.581, 2.420) (0.617) | 1.432 (0.636, 3.225) (0.361) | 0.406 |
| **Model 3 [OR (95% CI) (P-value)]** | Referent | 1.529 (0.732, 3.194) (0.239) | 1.100 (0.542, 2.230) (0.779) | 1.255 (0.566, 2.784) (0.552) | 0.773 |
| **Flavones** |  |  |  |  |  |
| **Range (mg/day)** | [0, 0.155] | (0.155, 0.505] | (0.505, 1.265] | (1.265, 72.825] |  |
| **Model 1 [OR (95% CI) (P-value)]** | Referent | 0.833 (0.336, 2.067) (0.669) | 0.944 (0.510, 1.747) (0.843) | 0.889 (0.314, 2.516) (0.809) | 0.864 |
| **Model 2 [OR (95% CI) (P-value)]** | Referent | 0.863 (0.346, 2.154) (0.736) | 1.024 (0.531, 1.977) (0.939) | 1.037 (0.360, 2.982) (0.943) | 0.851 |
| **Model 3 [OR (95% CI) (P-value)]** | Referent | 0.877 (0.361, 2.133) (0.758) | 1.101 (0.575, 2.106) (0.756) | 0.834 (0.267, 2.599) (0.738) | 0.847 |
| **Flavonols** |  |  |  |  |  |
| **Range (mg/day)** | [0, 6.819] | (6.819, 12.535] | (12.535, 22.669] | (22.669, 207.145] |  |
| **Model 1 [OR (95% CI) (P-value)]** | Referent | 1.540 (0.976, 2.429) (0.061) | 0.787 (0.474, 1.308) (0.325) | 1.539 (0.831, 2.848) (0.153) | 0.407 |
| **Model 2 [OR (95% CI) (P-value)]** | Referent | 1.537 (0.978, 2.417) (0.061) | 0.843 (0.487, 1.460) (0.517) | 1.735 (0.951, 3.164) (0.070) | 0.231 |
| **Model 3 [OR (95% CI) (P-value)]** | Referent | 1.521 (0.951, 2.433) (0.076) | 0.754 (0.388, 1.468) (0.381) | 1.443 (0.827, 2.516) (0.181) | 0.540 |

**Notes: OR, odds ratio; 95% CI, 95% confidence interval. Model 1: No covariates were adjusted. Model 2: Age, sex, race, education level, PIR, BMI, smoking status, alcohol use were adjusted. Model 3: Age, sex, race, education level, PIR, BMI, smoking status, alcohol use, diabetes mellitus, hypertension, hyperlipidemia, HEI-2015 scores, total energy, dietary intakes of protein, saturated fat, fiber, carbohydrates, polyunsaturated fat and physical activity were adjusted.**
